# Recent Advances in Basic and Clinical Colorectal Cancer Research

**DOI:** 10.3390/cancers18111834

**Published:** 2026-06-03

**Authors:** Seiichi Shinji, Tomio Arai

**Affiliations:** 1 Department of Gastrointestinal and Hepato-Biliary-Pancreatic Surgery, Nippon Medical School, 1-1-5 Sendagi, Bunkyo-ku, Tokyo 113-8603, Japan; 2 Department of Pathology, Tokyo Metropolitan Institute for Geriatrics and Gerontology, 35-2 Sakae-cho, Itabashi-ku, Tokyo 173-0015, Japan

## 1. Introduction

Colorectal cancer remains the third most commonly diagnosed malignancy and the second leading cause of cancer-related death worldwide [1]. Despite meaningful improvements in screening, surgical technique, and systemic therapy over the past two decades, outcomes in advanced and metastatic disease remain suboptimal, and the global incidence of early-onset CRC—diagnosed before the age of 50—continues to rise at an alarming rate [2,3]. Bridging the gap between laboratory discovery and bedside application therefore demands not only scientific breadth but also methodological innovation.

This Special Issue was conceived to address precisely that challenge. By bringing together contributions from basic scientists, translational researchers, and clinician-investigators across multiple continents, we sought to capture the breadth of contemporary CRC research while maintaining a clear translational focus. The twelve papers published herein span the spectrum from fundamental models of crypt cell biology to multicenter robotic surgical series, from the immunological intricacies of mismatch-repair-deficient tumors to the environmental sustainability of the operating theater. In this closing editorial, we synthesize the key findings across three overarching thematic areas and highlight directions for future investigation.

## 2. Early Detection, Risk Stratification, and Clinical Characterization

Two original articles address the clinical front line of CRC management—the challenge of identifying significant pathology before it becomes advanced disease, and the characterization of a patient population whose epidemiological profile is rapidly evolving.

Hijos-Mallada et al. evaluated a qualitative point-of-care (POC) fecal test combining four biomarkers—hemoglobin, transferrin, calprotectin, and lactoferrin—in a prospective cohort of 571 symptomatic patients undergoing colonoscopy. The POC test demonstrated a negative predictive value of 94.8% for significant pathology and 100% for CRC or inflammatory bowel disease when all four markers were negative, a finding with direct implications for triage. In an era of constrained endoscopic capacity, the ability to safely defer colonoscopy in more than one-third of symptomatic patients without missing a cancer represents a meaningful clinical advance. The authors also identified a high-risk positive pattern—all four markers positive—present in 6% of patients, of whom 70.6% had CRC or inflammatory bowel disease, underscoring the potential for bidirectional risk stratification from a single non-invasive test. Future work integrating such POC platforms into formal diagnostic algorithms and electronic health records will be essential to translate these operating characteristics into real-world benefit.

Akimoto et al. provide a granular clinicopathological characterization of endoscopically resected colorectal neoplasia in younger adults at a high-volume Japanese academic center. Analyzing 1299 patients with 3399 lesions, the authors demonstrate that early-onset neoplasia (diagnosed before age 50) exhibits a left-sided predominance, greater pedunculated morphology, larger lesion size, and a higher prevalence of advanced adenoma compared with later-onset cases—findings that could not be attributed to hereditary polyposis alone. These observations suggest that the biology of sporadic early-onset CRC may be genuinely distinct from that of its later-onset counterpart, supporting calls for tailored surveillance strategies and revised screening age thresholds. The data also provide a robust Japanese reference dataset at a time when most early-onset CRC epidemiology has been generated in Western cohorts.

## 3. Tumor Biology, Experimental Models, and Therapeutic Targeting

The scientific core of this Special Issue comprises six original articles and two review papers that illuminate CRC biology across multiple experimental platforms, from mathematical models of crypt dynamics to patient-derived organoids and three-dimensional bioprinting.

Boman et al. introduce a compelling theoretical framework for colon tumorigenesis rooted in the concept of autocatalytic tissue renewal. Using a system of nonlinear differential equations to model crypt cell population dynamics, the authors demonstrate that APC mutation decreases the rate of tissue renewal-based cell polymerization, creating a rate-limiting step that expands the proliferative cell population and promotes epithelial disorganization. Applied to familial adenomatous polyposis (FAP), this mathematical model recapitulates the premalignant phenotype with striking fidelity and offers a mechanism-based explanation for adenoma morphogenesis that transcends specific molecular targets. The framework opens avenues for identifying upstream therapeutic interventions targeting the kinetics of tissue renewal rather than individual oncogenic pathways.

Two complementary papers from McGuckin, Forraz, and colleagues advance the frontier of three-dimensional (3D) bioprinting as a platform for modeling CRC and its metastases. Their 2023 report described the first long-term (approximately six months) maintenance of patient-derived 3D bioprinted colorectal tumor models, enabling longitudinal studies of tumor evolution and oncolytic virus screening at an unprecedented temporal scale. The 2025 follow-up paper extended this approach to hepatic metastasis modeling and demonstrated the delivery of an oncolytic viral chemotherapeutic agent directly to tumor cells within bioprinted liver metastasis constructs. Taken together, these studies establish bioprinted patient-derived models as a viable next-generation screening platform, complementing—and, in key respects, surpassing—conventional 2D cell line and organoid systems for the evaluation of complex therapeutic modalities.

Tozaki et al. report a synthetic lethality strategy targeting the DNA damage response in CRC, combining inhibitors of ataxia telangiectasia-mutated kinase (ATM) and checkpoint kinase 1 (Chk1). The combination produced synergistic antitumor effects at individual subtherapeutic doses in vitro, mechanistically driven by unrestrained cyclin-dependent kinase 1 activation and resultant apoptosis. Importantly, robust activity was confirmed in a syngeneic in vivo model, strengthening the translational relevance of the observations. This work adds to a growing literature on DNA damage response pathway co-targeting in CRC and provides a rationale for a clinical investigation of ATM/Chk1 inhibitor combinations in patients whose tumors harbor relevant alterations.

Sampaio-Ribeiro et al. characterize the innate immune landscape of the tumor microenvironment in CRC liver metastases using multi-parameter flow cytometry of paired tumor and non-tumor biopsy specimens from 47 patients. The study identifies elevated PD-L1 (CD274) expression on monocyte subsets in tumor versus non-tumor tissue and demonstrates that the desmoplastic growth pattern is associated with reduced PD-L1 and CD206 expression—a phenotype correlated with improved disease-free survival. These findings position monocyte-expressed immune checkpoint molecules as potential biomarkers and therapeutic targets in the metastatic setting and provide mechanistic grounding for combinatorial immunotherapy strategies that target both monocyte polarization and canonical T-cell checkpoint pathways.

Son et al. evaluate fluorescence lymph node mapping (FLNM) using indocyanine green (ICG) in a prospective case–control study of laparoscopic right hemicolectomy. Endoscopic submucosal ICG injection prior to surgery enabled real-time visualization of D3 lymphatic drainage pathways, resulting in a significantly higher yield of harvested D3 lymph nodes in the FLNM group. Critically, the proportion of D3 lymph node metastases was higher in stage III patients in the FLNM group, suggesting that conventional surgical dissection may systematically underestimate the metastatic burden. FLNM therefore emerges as a technically accessible strategy for intraoperative surgical quality assurance that warrants evaluation in broader multicenter trials.

The two review articles in this thematic cluster provide comprehensive surveys of foundational importance to the field. Al-Kabani et al. offer a critical appraisal of experimental CRC models across the full spectrum—from 2D cell lines and spheroids to patient-derived organoids, genetically engineered mouse models, zebrafish, organ-on-chip platforms, humanized mouse avatars, CRISPR-engineered systems, and AI-augmented simulations. The authors provide a systematic framework for matching model capabilities to specific research questions. The central message is one of strategic complementarity: no single model captures the full complexity of human CRC, but thoughtful integration of multiple platforms maximizes translational validity.

Heregger et al. review the mechanisms underlying resistance to immune checkpoint inhibitors (ICIs) in mismatch-repair-deficient (dMMR)/microsatellite instability-high (MSI-H) CRC, a subtype in which the therapeutic promise of immunotherapy is most clearly established yet still incompletely realized. The authors systematically address tumor-intrinsic factors—alterations in the antigen-presenting machinery, WNT/β-catenin signaling, interferon-γ pathway mutations, and TGF-β-mediated immunosuppression—alongside extrinsic determinants including variable immune checkpoint expression, gut microbiota composition, and the Immunoscore. The review also highlights diagnostic pitfalls in MSI-H testing and the heterogeneity between Lynch syndrome-associated and sporadic MSI-H disease. With approximately 30% of dMMR/MSI-H patients failing to respond to first-line ICI therapy, this mechanistic taxonomy provides an essential roadmap for the rational design of combination strategies.

## 4. Surgical Innovation, Artificial Intelligence, and Sustainable Practice

Two papers in this Special Issue address the transformation of the operative environment through technology, representing the increasingly prominent intersection of surgery, engineering, and sustainability science.

Azevedo et al. report a multicenter retrospective analysis of 501 consecutive robotic colorectal cancer resections across five specialist units, using the textbook oncological outcome (TOO) as a composite quality metric. A TOO—defined as no conversion to open, no Clavien–Dindo grade ≥3 complication, length of stay ≤14 days, no 30-day readmission, no 30-day mortality, and R0 resection—was achieved in 77.4% of patients, comparing favorably with published benchmarks for both laparoscopic and open colorectal surgery. Abdominoperineal resection was the sole independent risk factor for TOO failure. These data validate the TOO framework as a patient-centric outcome measure for robotic colorectal surgery and provide a performance benchmark against which individual units and emerging programs can be evaluated.

Iwai, Shinji, and colleagues address the sustainability crisis confronting modern surgery through a dual lens: the application of intraoperative AI navigation systems and the adoption of reusable energy devices in laparoscopic colorectal surgery. Surgical AI systems capable of real-time anatomical structure recognition—connective tissue planes, ureters, nerves, and vascular structures—offer three compounding benefits: enhanced surgical precision, facilitation of task-shifting to non-physician scrub personnel, and accelerated training of junior surgeons through objective, AI-validated dissection plane feedback. The authors’ institutional data on the Reusable Energy Device Laparoscopic-Assisted Colectomy (RE-LAC) technique demonstrated non-inferior operative time and blood loss compared with conventional single-use devices across 52 consecutive patients, while aligning with the United Nations Sustainable Development Goals for health, industry, responsible consumption, and climate action. This paper exemplifies a vision of surgical sustainability in which technological advancement and environmental stewardship are complementary rather than competing imperatives.

## 5. Cross-Cutting Themes and Future Directions

Reading the twelve contributions as a whole, several cross-cutting themes emerge that are likely to define the trajectory of CRC research in the coming decade.

First, the integration of artificial intelligence permeates this Special Issue at multiple levels—from AI-augmented tumor models and organoid platforms to intraoperative anatomical navigation and the computational modeling of crypt dynamics. As these applications mature, their convergence with large-scale molecular profiling and clinical data will accelerate the identification of actionable biomarkers and personalize therapeutic decision-making in ways that were not previously achievable.

Second, the tumor microenvironment emerges as a unifying thread connecting immunotherapy resistance, liver metastasis biology, and experimental model design. The shift from tumor-cell-centric to ecosystem-level thinking demands experimental platforms that faithfully recapitulate the spatial and cellular complexity of the microenvironment—a requirement that is driving the maturation of organoid co-culture systems, organ-on-chip technologies, and humanized mouse avatars.

Third, the clinical challenge of early-onset CRC demands urgent attention. The distinct anatomical and pathological profile of neoplasia in younger adults suggests that current surveillance algorithms, designed for an older population, may be inadequate. Prospective studies examining the molecular characteristics, germline landscape, and optimal surveillance strategies for early-onset CRC represent a high-priority research agenda.

Finally, the surgical papers in this collection reflect a broader maturation of minimally invasive colorectal surgery. Robotic platforms are demonstrating consistent oncological quality across diverse institutional settings, while fluorescence-guided lymph node mapping and AI-assisted anatomical navigation are progressively reducing the technical variability that has historically limited the reproducibility of surgical outcomes. The convergence of these technologies holds the prospect of a new standard of surgical care in which quality is systematically assured rather than operator-dependent.

## 6. Conclusions

This Special Issue captures colorectal cancer research at an inflection point. The contributions collected here are united by a commitment to translational relevance: each, in its own way, moves the field closer to the personalized, technology-enabled, and environmentally responsible care that patients deserve. We are grateful to all authors for their outstanding contributions, to the reviewers whose critical insights strengthened each manuscript, and to the editorial team of *Cancers* for their support throughout this Special Issue.

We trust that the readers of this collection will find in it both scientific stimulation and practical guidance—and that the questions it raises will catalyze the next generation of discoveries in colorectal cancer research.

## Data Availability

Not applicable.
